# Use of electronic medical records and biomarkers to manage risk and resource efficiencies

**DOI:** 10.1080/20018525.2017.1293386

**Published:** 2017-03-14

**Authors:** Dermot Ryan, John Blakey, Alison Chisholm, David Price, Mike Thomas, Björn Ställberg, Karin Lisspers, Janwillem W. H. Kocks, on behalf of the Respiratory Effectiveness Group

**Affiliations:** ^a^ Usher Institute of Population Health Sciences and Informatics, University of Edinburgh, Edinburgh, UK; ^b^ Respiratory Effectiveness Group, Cambridge, UK; ^c^ Centre for Academic Primary Care, University of Aberdeen, Aberdeen, UK; ^d^ Observational and Pragmatic Research Institute, Singapore​​​​; ^e^ Clinical Sciences, Liverpool School of Tropical Medicine, and Respiratory Medicine, Royal Liverpool Hospital, Liverpool, UK; ^f^ Primary Care and Population Sciences, Faculty of Medicine, University of Southampton, Southampton, UK; ^g^ Department of Public Health and Caring Science, Family Medicine and Preventive Medicine, Uppsala University, Uppsala, Sweden; ^h^ Department of General Practice and Research Institute for Asthma and COPD (GRIAC), University of Groningen, University Medical Center Groningen, Groningen, the Netherlands

**Keywords:** Electronic medical record (EMR), electronic health record (EHR), database, asthma, biomarker, primary care​​​​, CDSS, big data

## Abstract

The migration from paper to electronic medical records (EMRs) was motivated by the administrative need to record, retrieve and process increasing amounts of clinical data in the 1980s. In the intervening period, there has been growing recognition of the potential of such records for achieving care efficiencies, informing clinical decision making and real-life research. EMRs can be used to characterise patient groups, management approaches and differential outcomes. Characterisation can also help with identification of potential biomarkers for future risk determination and likely treatment response. The future heralds even greater opportunities through integration of clinical records and a range of technology-based solutions within a more complete electronic health record (EHR). Through application of algorithms based on identified risk predictors and disease determinants, clinical records could also be used to enable risk stratification of patients to optimise targeted interventions, conserving resources to achieve individual patient and system-wide benefit. In this review, we reflect on the evolution of the EMR and EHR and discuss current and emerging opportunities, particularly with respect to biomarkers and targeting of innovative biologic interventions. We also consider some of the critical issues associated with realising the potential of the EHR as a clinical aid and research tool in an age of emerging technologies.​​​​

## The origins of electronic medical records

​​​​Electronic medical records (EMRs) were first introduced in the 1980s, motivated by the need for more sophisticated data management in the face of ever more (and more complex) medical information.[1,2] The increasing volume of clinical and laboratory data available required the development of an infrastructure that facilitated data capture, storage and searchability. In parallel, escalating healthcare costs required clinical events to be time- and date-stamped using standardised disease and drug coding hierarchies to facilitate reimbursement administration and to track healthcare utilisation and budgets.

The pace of transition from paper to electronic medical records differed between countries, led by more developed healthcare systems but also catalysed by regulation and/or financial incentives. In the USA, a 2014 federal mandate required all public and private healthcare providers and other eligible professionals to adopt and demonstrate ‘meaningful use’ of EMRs in order to maintain their existing Medicaid and Medicare reimbursement levels.[3] ‘Meaningful use’ was targeted at their potential to improve healthcare quality, safety, efficiency and coordination, and patient and family engagement with a view to reducing healthcare disparities and improving public health. Although not so expressly mandated elsewhere, early government incentives around EMRs saw rapid, widespread adoption in the UK and an ostensibly ‘paper-free’ approach to clinical data recording within primary care by the turn of the millennium. Although there was no financial incentive attached, EMRs were also adopted early in Sweden; implemented within primary care in the mid-1990s and in secondary care at the turn of the millennium.

Where the migration from paper to electronic medical records has occurred, EMRs have not only provided a more efficient means of recording patient data and an improved infrastructure for billing, they have also provided unprecedented opportunities to improve clinical management and for clinical research.

## Current use – clinical management and resource efficiencies

Electronic medical records contain a wide range of structured, routinely recorded care clinical and demographic data that can be used to improve understanding of population and disease profiles and of current practice. Characterisation can be applied to individual practices or more broadly: at the regional, national or even international level. Cross-sectional analysis of EMRs can help to quantify and characterise disease course and prevalence, healthcare resource use and routine care management approaches (e.g. service audit and evaluation of guideline implementation). Such data can assist with effective, efficient resource planning and care provision by providing a baseline against which change can be measured. They also provide the opportunity to identify examples of both optimal and suboptimal practice and over- and under-resourcing, which can help to predict and mitigate against future system pressures and shortfalls in care.[4,6]

Longitudinal analysis of EMRs can be used to further characterise care pathways (e.g. patients’ diagnostic journeys) [7], disease progression and to evaluate the (comparative) safety and effectiveness of interventions. Comparative effectiveness studies using routine care data offers a means of evaluating real-life treatment outcomes and differential treatment responses across patient subpopulations as a complement to idealised clinical trial efficacy outcomes.[5]

The creation of these very large datasets coupled with the increasing capability of machine learning approaches could be used to develop dynamic clinical decision support systems (CDSS) to optimise clinical benefit and minimise patient risk. In the Netherlands, the majority of EMRs incorporate a decision support tool developed in collaboration with the Dutch College of General Practitioners. It has been rolled out across a range of chronic disease areas since 2009 and is widely accepted by general practitioners and practice nurses.[8] A recent evaluation of the system, however, found that although the CDSS is positively viewed it has limited impact on clinicians’ behaviour. When questioned about the reasons for their low implementation of the system’s care recommendations, user feedback revealed a lack of awareness of the system’s functionality, limited belief in its relevance and value and perceived challenges in integrating the recommendations into daily practice.[9] These barriers to uptake are the cautionary counter to the potential of the EMR: in order to realise the potential of EMR-based CDSS, there is a need for clear planning and co-design with healthcare professionals to ensure they are valuable and practical tools.

## Emerging opportunities: risk reduction and biomarkers

**Figure 1.**
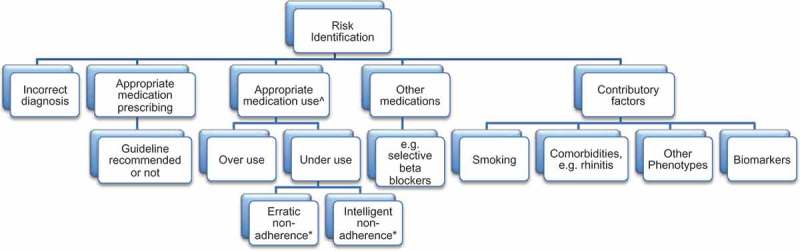
Variables available within routine primary care EMRs that can be used to explore future risk in respiratory disease. Notes: ^Inhaler technique/handling errors are also important, but device issues are not routinely captured in primary care records. *World Health Organization (WHO) adherence categories.[14]

Beyond their potential for general patient and practice characterisation, there has also been a growing trend to use EMRs (Figure 1) to identify ‘at-risk’ clinical practices and patient characteristics.[10–12] Interrogation of EMRs has also revealed the potential value of routinely recorded data to identify and validate the use of existing and exploratory biomarkers (e.g. blood eosinophils, FeNO [existing]; exhaled breath condensate markers [proposed]).[13] The integration of biomarker and existing clinical data may help to tailor diagnostic and management decisions to individual patients and their needs and to develop a more dynamic and responsive CDSS.

### Biomarkers within obstructive airways disease diagnosis

In the absence of a single diagnostic test for asthma, diagnosis involves a combination of clinical assessment (history taking, objective measurements) and ‘clinical judgment’.[15] The more clinical evidence available, the more accurate the diagnostic accuracy and better informed the selection of treatment options. By contrast the diagnosis of COPD is defined by the presence of a threshold level of airway obstruction in conjunction with characteristic symptoms.[16] Disease features (e.g. symptom scores and exacerbation frequency) have been introduced as additional dimensions to help guide and direct COPD therapy.[17] Thus routinely recorded biomarkers – objectively measured and evaluated characteristics that can indicate normal biological or pathogenic processes and/or potential response to a pharmacological intervention [18] – may hold the potential to further support diagnostic and optimum therapeutic decision making.

Almost a dozen potential biomarkers have (so far) been proposed in asthma; see Table 1.[19,20] Nine of the biomarkers currently proposed are potentially viable for evaluation in observational studies, but only three via retrospective observational studies using routinely collected primary care EMRs. These are: complete blood counts (CBC), particularly blood eosinophil count, fractional exhaled nitric oxide (FeNO), and total/allergen-specific immunoglobulin E (IgE).

**Table 1.** List of biomarkers of potential use in asthma; those in bold are the most likely candidates for interrogation using routine care EMRs.

	Characterisation of study populations for prospective clinical trials (i.e. baseline information)	Prospective clinical trial efficacy/effectiveness outcomes	Observational study outcomes*
**Core outcomes**	Serologic multi-allergen screen (IgE) to define atopic status (also for observational studies)	None	None
**Supplemental outcomes**	**Feno**	**Feno**	**Feno**
	Sputum	Sputum	Sputum
	**CBC (total eosinophils)**	**CBC (total eosinophils)**	**CBC (total eosinophils)**
	**Total IgE**	**Total IgE**	**Total IgE**
	**Allergen-specific IgE**	**Allergen-specific IgE**	**Allergen-specific IgE**
	Urinary LTE_4_	Urinary LTE_4_	Urinary LTE_4_
**Emerging outcomes**	Allergen skin prick testing	Allergen skin prick testing	
	Sputum neutrophils and analyses	Sputum neutrophils and analyses	Sputum neutrophils and analyses
	Airway imaging	Airway imaging	Airway imaging
	Exhaled breath condensate markers	Exhaled breath condensate markers	
	Discovery through genetics and genomics	Discovery through genetics and genomics	Discovery through genetics and genomics

*Observational study designs include cohort, case-control, cross-sectional, retrospective reviews; genome-wide association studies (GWAS) and secondary analysis of existing data. Some measures may not be available in studies using previously collected data.

#### Eosinophils

Complete blood counts provide a clear example of the potential for biomarker identification and patient risk profiling using routinely recorded EMRs.

Asthma is a condition frequently driven by eosinophilic airways inflammation.[21] The extent of eosinophillic inflammation present in patients with asthma can be detected in a variety of ways, among them induced sputum analysis. Raised sputum eosinophil count (sputum eosinophilia) has been identified as a marker of future asthma risk, with studies suggesting that sequential sputum eosinophil assessment could be used as a model for managing patient care.[22] Yet the technical skill involved in sputum induction and analysis are barriers to its use in routine care. Blood eosinophils, in contrast, are relatively easy and inexpensive to assess in primary care. Indeed, as blood tests are used in primary care for a number of reasons (e.g. often as a means of general exploratory investigation in response to reported fatigue) CBC and blood eosinophil data are present in a large number of patients’ records. Not only are blood eosinophils relatively easy to evaluate, they also appear to correlate well with sputum eosinophilia [23] and to be responsive to inhaled corticosteroid (ICS) therapy.[24] In addition, an observational study using routine care EMRs concluded that UK patients with asthma and blood eosinophil counts greater than 400 cells per μl experience more severe exacerbations and have poorer asthma control than those with lower blood eosinophil counts and reported a count–response relationship between blood eosinophil counts and asthma-related outcomes.[11] Similar to the dose ranging efficacy and safety with mepolizumab in severe asthma (DREAM) study,[12] there was a clear association seen between increasing eosinophil count and both poorer asthma control and higher rates of exacerbation. Thus there is compelling evidence to suggest blood eosinophilia may predict response to ICS therapy in asthma, but currently insufficient data available to inform dose- and count-specific recommendations.

In COPD, interest in eosinophil count as a potential biomarker has centred on its potential to predict ICS treatment response and reduce ICS over treatment in a disease that is ostensibly unresponsive to steroid treatment.[25] Recent studies suggest that ICS withdrawal may be a safe and effective strategy in COPD,[26] but there is a belief that a significant number of patients may derive some degree of benefit from ICS.[27] Thus tools are required to help differentiate such patients from those who may benefit from steroid tapering strategies. As evidence of eosinophil presence in the airways indicates the presence of steroid-responsive disease, confirmatory peripheral markers such as elevated blood eosinophils may help identify patients who could benefit from steroid treatment. There are now analyses that support this approach,[28,29] but a widely accessible and affordable point-of-care tool to identify COPD patients with eosinophilic inflammation with a view to informing management approaches remains a future aspiration and more likely the preserve of specialist care in the short term.

#### Fractional exhaled nitric oxide

Recent evidence suggests FeNO may be a suitable alternative to blood eosinophils in identifying pulmonary eosinophilic inflammation in adults,[30] as it offers the potential to inform the diagnosis and management of obstructive lung disease, most notably asthma.[31,32] FeNO offers the benefit of being a non-invasive and reproducible assessment that can be easily carried out in primary care (in minutes) using hand-held meters, although there are some limitations when used in patients who smoke.[33] EMR studies have largely been thwarted by poor uptake of FeNO assessment in clinical practice. This may reflect a lack of understanding of its potential utility and financial concerns around upfront cost of FeNO machines and/or a lack of universal reimbursement despite evidence demonstrating its affordability in a routine primary care setting.[34]

**Figure 2.**
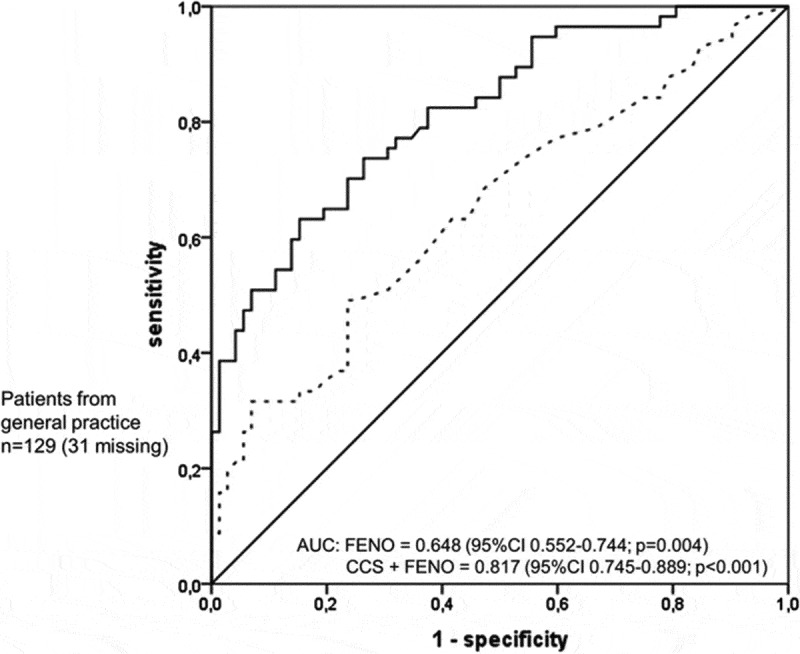
Receiver operating characteristic curves of fractional exhaled nitric oxide and clinical signs and symptoms. Adding clinical symptom scores to FeNO can help to confirm asthma in patients for whom the AUC is significantly shifted to the left. Reproduced from BJM Open, Schneider A, Wagenpfeil G, Jörres RA, Wagenpfeil S. 5:e009676, 2015 with permission from BMJ Publishing Group Ltd.[36]​​​​

The American Thoracic Society [35] offers guidance for the interpretation and clinical use of FeNO, outlining low (≤25 ppb) and high (≥50 ppb) thresholds below which inflammatory airways disease can be ruled out and above which eosinophilic inflammation is likely. Where further guidance is required, particularly in the intermediate range (26–49 ppb), there may be value in combining FeNO readings with clinical symptom scores to help substantiate a diagnosis of asthma, particularly to ‘rule-in’ asthma in the primary care environment (see Figure 2).[36]

The most recent Cochrane review on the use of FeNo recommends its selective use in patients who have frequent asthma exacerbations.[37] Yet the related literature is evolving rapidly and clear guidance is still awaited on the optimal use of FeNO as a practical tool in wider asthma management.[38–40] The real-life case studies featured in Figure 3 provide some examples of how FeNO could be used to guide asthma diagnosis and subsequent assessment and monitoring within routine primary care.

**Figure:**
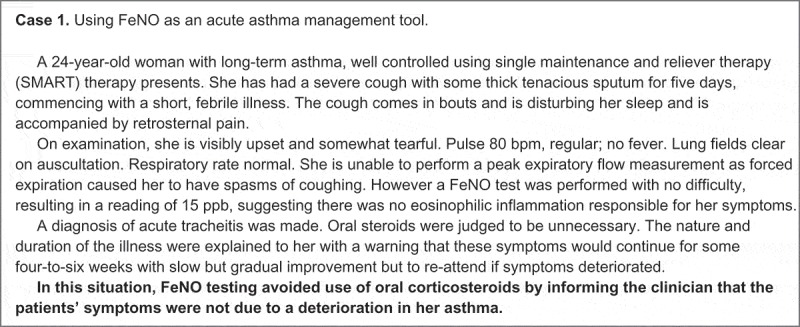

**Figure 3.**
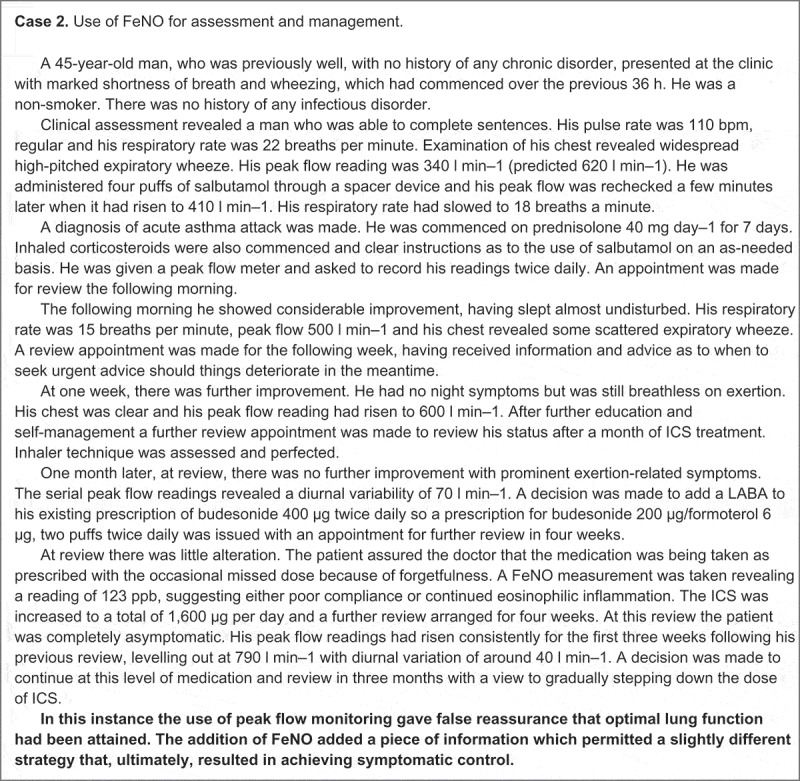
Illustrative case examples of how FeNO and blood eosinophils can be used alone, or in combination, to guide diagnostic and clinical decision making in routine primary care. They are drawn directly from UK clinical practice and are not intended to suggest a paradigm of clinical perfection, but serve to exemplify the use of evidence applied to an individual patient’s clinical need. The cases have been abbreviated and simplified to maintain patient confidentiality (without adaptation of the clinical content).​​​​

**Figure 3.**
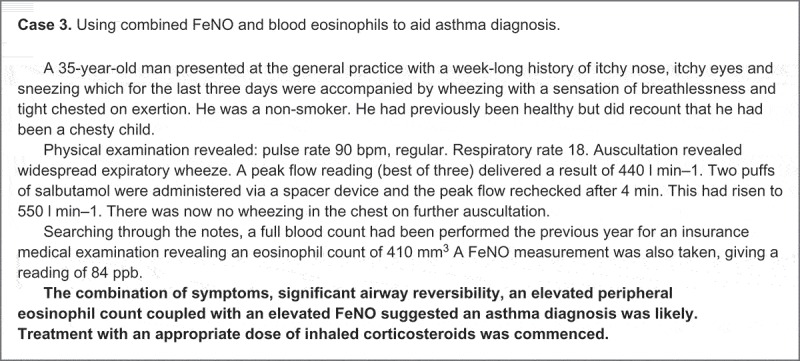
Continued.

Within COPD, the role of FeNO as a clinical management tool is less well explored, but there are preliminary data to suggest intermediate (>25 ppb) and high (>50 ppb) levels of FeNO could aid in the identification of patients with a mixed asthma-COPD phenotype.[41]

#### Serum immunoglubulin e (IgE)

IgE has to date infrequently been assessed in primary care, limiting opportunities to use EMR to explore the potential of total and specific IgE (SIgE) measurements. The SIgE landscape may be on the verge of change, however: the Dutch national asthma guidelines have recently recommended SIgE assessment to be conducted in all patients with suspected allergies, which will result in an inevitable increase in data points and research opportunities.[42]

The recent American Academy and American College of Asthma, Allergy and Immunology joint statement on allergy testing notes the limited contribution of total serum IgE estimation to allergy diagnosis.[43] The joint statement also advises that interpretation of specific IgE, as with skin tests, requires correlation with the history, physical examination, and, in some cases, symptoms directly observed after exposure to allergens, a view shared by the European Academy of Allergy and Clinical Immunology.[44] The true value of specific IgE as a potential biomarker, or possible proxy for care quality,[44] awaits evolution of the single-system EMR to more complete electronic health recording that integrates data from a range of clinical and environmental sources. The increasing number of specific IgE data points collected in the Netherlands may offer a means of confirming the utility of this approach.

#### Combined biomarkers

As with all clinical approaches, improved understanding of the clinical situation is achieved by piecing together different information sources and integrating complementary data. While routinely captured biomarkers can be used in isolation to predict future exacerbation risk, they can also be used in combination with other routinely collected data to create more powerful, composite metrics for predicting future risk.[12,45,46, 47]

In addition, there is the potential promise of greater clinical insight through combining FeNO and blood eosinophil values to generate a ‘composite inflammatory biomarker’. Both are markers of local and systemic eosinophilic inflammation and both are elevated in patients with asthma.[47–49] Using data from the National Health and Nutrition Examination Survey (NHANES) cohort, Malinovschi et al. explored the associations between blood eosinophil and wheeze, asthma diagnosis, and asthma events and between FeNO and wheeze, asthma diagnosis and events in a random sample of more than 12,000 patients.[50] Elevated (intermediate or high) FeNO values and intermediate or high blood eosinophil values were independently associated with all three outcomes. This independent association with wheeze, asthma diagnosis and asthma events suggests that FeNO and blood eosinophils may measure different aspects of inflammation (both local and systemic) and that their use in combination could improve phenotyping in patients with asthma.

Further work is required to establish the additive benefit of combining FeNO with blood eosinophils in higher-step asthma patients. Questions persist as to whether raised FeNO and blood eosinophil count may be markers of a persistent inflammatory phenotype that does not respond to ICS therapy and whether they could be used to identify patients who will benefit from specialist referral and possible biological therapy. The role of retrospective EMRs in the validation of these findings is currently limited by the dearth of FeNO data in routine care records, but existing EMRs could be used to identify patients with complete blood eosinophil records for prospective FeNO data collection. Supplementation of routine care EMRs with prospectively collected FeNO data could be a feasible and low-cost approach to further investigating blood eosinophil levels and FeNO as a composite biomarker in the diagnosis and management of asthma. Figure 3, case 3 illustrates how the UK authors of this paper have utilised FeNO and eosinophil data in combination, to guide clinical decision making in their own practice settings.

Other potential candidates for composite asthma biomarkers include total serum IgE and blood eosinophils to predict response to omalizumab. A *post-hoc* analysis of the INNOVATE Trial [51] examined the impact of omalizumab treatment (vs. placebo) on exacerbation rates and health-related quality of life in patients with severe allergic asthma (SAA), stratified by peripheral blood eosinophils and serum IgE.[52] The investigators concluded that patient subgroups with a combination of increased serum IgE and blood eosinophils might experience greater clinical benefit. Pooling routine EMRs at a national (and even international) level could help to generate hypotheses and to power further exploratory studies in SAA, such as to test the hypothesis that combining serum IgE with blood eosinophils may offer additional benefit in identifying patients with a mixed asthma-COPD.[53,54]

## The future of EHRs

The preceding sections have largely discussed the analysis potential of EMRs gathered and reported in a single setting such as primary care. Many systems now incorporate an ability to share information between providers – a move toward genuinely more extensive, integrated electronic health records (EHRs).[55] A more complete picture of an individual’s health requires additional information than can be manually entered by clinicians during brief consultations. The next stage of development for EHRs is the routine incorporation of data from connected devices.

The exponential increase in available processing power and accompanying reduction in the size and cost of sensor technology has brought about a formidable change in the use of everyday items, most notably mobile telephones. In this context, there are now ‘smart’ versions of many common inhalers and of peak flow meters and spirometers. These innovations permit real-time assessment of medication use (concordance and beta-agonist consumption) and inhaler technique. Such data can be placed in the context of combining a fine-grain symptom record, incorporating peak flow readings, location and activity, environmental data such as pollen count [56] or pollution levels, ultimately validating their inclusion or exclusion within a CDSS.

Introducing data from such systems into the EHR offers the potential for semi-automated standard action plans, via a CDSS, reducing the risk of inappropriate treatment. Relevant healthcare advice (such as inhaler technique guidance) could be offered through a personalised interface such as an integrated mobile phone app. Clinicians may favour using such a system to provide inhaler technique advice based on robust existing evidence,[57,58] but it is uncertain whether this specific approach will engage people with asthma. The EU-funded MyAirCoach project is an ambitious collaborative initiative that aims to deliver an evidence base for this challenging area. The ambition of MyAirCoach is to integrate technology: ‘with a smart sensing infrastructure and clinical prediction models in order to provide personalised feedback to patients on how to manage their condition in their home or at work, without the need to have frequent face-to-face contact with healthcare professionals in the hospital or clinic.’[59]​​​​ The initial phases of MyAirCoach have focused on understanding end user preferences and attitudes, to begin to build an interface that is intuitive for patients of different ages and backgrounds, while also increasing their involvement in the healthcare process. MyAirCoach also aims to facilitate healthcare professionals’ access to a detailed and accurate picture of the patient’s health state when they are away from the clinic environment. This will allow a more objective assessment of an individual’s current status and the evolution of their condition, and better inform treatment decisions. Clinicians will soon have remote warning of individuals with high beta-agonist use and /or low peak flow, with an early alert carrying the potential for intervention to avert a serious adverse outcome. Objective data from smart inhalers on adherence and technique already informs the decision to step up treatment in severe asthma centres (Refractory Asthma Stratification Programme [RASP]-UK [60]). The benefit to payors will be a move away from the inefficient annual review of airways disease to a more personalised, dynamic and responsive model with more efficient and targeted use of face-to-face consultations, offering the potential to reduce the number and duration of exacerbations and hospital admissions without expanding the current staffing resource.

**Figure 4.**
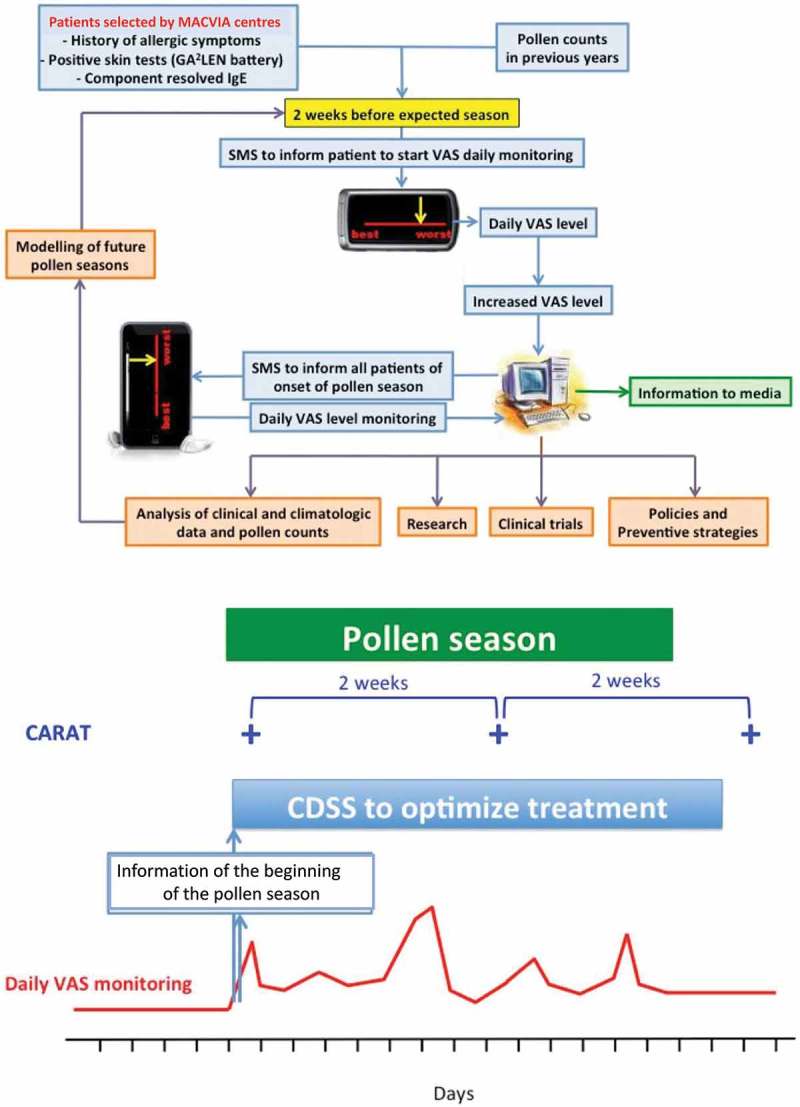
Examples of the MACVIA-ARIA Sentinel NetworK (MASK) to guide implementation of management interventions for patients with severe pollen allergies.[56] Reproduced with permission from John Wiley and Sons.​​​​

At the population level, integration of new data into the EHR may also enable identification and monitoring of sentinel networks. Using pollen allergy as an example, the networks would include highly sensitised and characterised patients with asthma and rhinitis whose condition can be easily triggered by rising pollen counts. By monitoring and identifying changes in control within sentinel groups it is feasible to forewarn the wider geo-localised population of recommended management approaches (e.g. trigger avoidance and/or pharmacological step up; see Figure 4).[56]

An integrated EHR containing standard clinical information from multiple healthcare records, new biomarker results, and behavioural and contextual data from connected devices remains the ultimate goal, a ‘big data’ scenario. This detailed description of an individual is likely to be a better predictor of future risk of adverse events, and of likely response to specified treatments. This ambition is being supported at the European level through the CONNECARE project which aims to develop, deploy and evaluate an adaptive integrated care system for chronic care management (including asthma and COPD) that will provide decision support for the adaptive management of personalised clinical pathways and will deliver tools to monitor patients’ activities and status, thus empowering them and providing them with recommendations to self-manage their condition.[61]

### Data quality and future proofing

We have presented the case and the potential of the EHR, but for these remarkable opportunities to become realities many significant practical challenges must be overcome. A full discussion of this area is beyond the scope of this review, but key aspects pertinent to EHR include data quality especially input and coding, and (from an emerging technologies perspective) data standards, visualisation, integration and analysis. The medical workforce will also need to be trained to understand, accept and utilise the opportunities offered and to co-produce software and connected devices.

Quality data are critical to the effective realisation of the care and resource efficiencies EHRs promise and to the calibre of research they can sustain. Data quality encapsulates a multitude of dimensions, among them: specificity of coding; scope nature of variables (categorical /continuous), completeness of coding; completeness of data and, when using the data to inform CDSS, the representativeness of the data.

Some of the issues around data quality can be illustrated through brief discussion of the role of the UK’s Quality and Outcomes Framework (QOF) on primary care EMRs in the UK. QOF was introduced in 2004 as part of the General Medical Services (GMS) contract for general practices [62] and governs reimbursement to practices for the provision of ‘quality care’ against certain outlined parameters. QOF incentivises practices to record various care activities within the EHR to ensure attainment of prescribed QOF criteria. In obstructive airways disease, this includes spirometric confirmation of COPD and performance of an annual review for anyone with asthma or COPD (i.e. two reviews for patients allocated a diagnostic code for both conditions). The influence of QOF on the EMR can be seen in terms of the more complete lung function records available for patients with a COPD since 2004 and (a degree of) ‘cleaning up’ of the diagnostic coding of asthma and COPD. Yet it also drives data collection around certain parameters (a ‘points for prizes’ mentality) and does not extend to all management aspects of all conditions. Still considering the UK, there are issues around data completeness as primary and secondary care EMRs remain largely discrete systems. Secondary care events are only captured in the primary care EMR as a result of manual input by general practitioners and are, as a result, systematically under-recorded. Data linkage approaches using anonymised patient identifiers to triangulate and integrate different data sources (hospital episode statistics, primary care, mortality records, etc.) to provide a more complete picture have achieved very limited success. The situation, again, varies between countries.

In some parts of Sweden, primary and secondary care use the same digital recording system, making it is possible for healthcare professionals to access a more complete record (including primary and secondary care data, laboratory data, medication lists and X-rays), which offers great benefit to clinical work. In several regions there is also a central database for spirometry results with shared access across primary and secondary care professionals. Swedish national healthcare quality assessment involves remote extraction and curation of certain parameters within disease-specific registers. These data are then used for research and to provide feedback to healthcare professionals with regards to benchmarking of their practice against national guidelines, priorities and quality indicator attainment.[63]

At an international level, pooling national datasets can power micro-stratification based on individual patient characteristics compared to matched controls and assist with the evaluation of incidence and prevalence of rare events and diseases. The benefits of international data linkage come with considerable challenges: navigating differing national legislations on data usage, normalising datasets across core standardised variables (and units) and aligning different coding systems (e.g. ICD-9 and 10, Read codes, SNOMED, ICPC). ​​​​Within the realms of what is legally permissible, integration is feasible if supported by application programming interfaces (APIs) and identification of key common variables critical to meaningful outcome evaluation, as demonstrated by the Uncovering and Noting Long-term Outcomes in COPD to enhance Knowledge (UNLOCK) Group.[64]

From an integrated EHR perspective, data from multiple types of smart inhaler or other connected technologies must be recorded following common definitions of core characteristics (e.g. medication adherence, lung function, control, exacerbations, healthcare resource utilisation). The data transferred from the devices will need to use a common API so that data can be pooled and integrated across sources. It should also be secure in transit and in a form that does not breach a patient’s wishes regarding privacy (e.g. geolocalisation). Standardisation will be difficult to achieve in the early phase of adoption where many small and medium enterprises are producing devices and software, combined political pressures and market forces should encourage longer-term planning by developers with greater engagement with established healthcare providers.

#### Barriers

**Figure 5.**
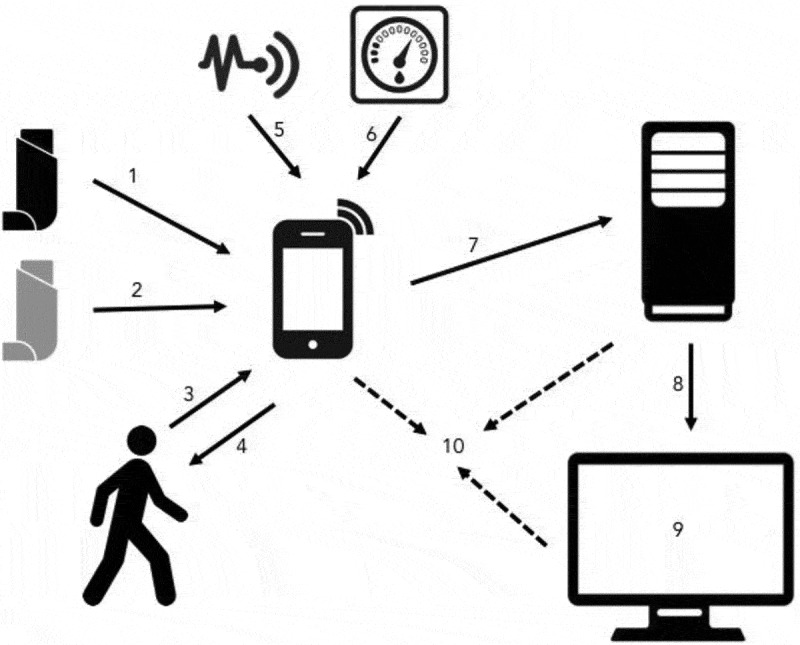
Illustration of areas where caution is required in the uptake of emerging healthcare technology based solutions.​​​​ Data from connected devices may require specific smartphone software (1) which may not be compatible with all devices (2), restricting treatment choice. Users may not enter symptom data regularly or accurately (3), and could be misled by feedback (4). Sensors may not be reliable (5) or accurate (6). Data transfer into secure clinical networks is not straightforward (7), and it needs to be correctly incorporated into individuals’ EHRs (8). Displaying large amounts of complex real-time data to inform clinical decision-making is challenging (9). At all stages, there is the perceived risk of data being gathered by third parties (10).

The respiratory community has much to gain from the implementation of electronic records, connected devices, and biomarkers. However, any significant change in clinical practice also carries risks of potential harm and cost. These risks are amplified when there are multiple aspects to the change, rapidly evolving new technologies are involved, and there is a step change in the amount of information available. Figure 5 illustrates key potential issues: there are already examples of problems such as relatively inaccurate sensors,[65] misleading user [66] or clinician feedback [67] and some users disengaging from remote monitoring.[68] Additional problems arise from providers acquiring different hardware or software solutions before interoperability standards are established. Retrospective resolution of such issues is possible and rewarding but time-consuming and costly: pro-active collaborative planning is preferable.

The recent Wachter Review outlines core principles for the transformation to a fully paperless healthcare service.[69] Alongside this, interoperability issues are beginning to be addressed by groups such as INTEROPen [70] and HL7 which bring together suppliers, providers and NHS digital information systems.

Further, the analysis and visualisation of these new data streams will pose challenges for clinicians coupled with a need to prevent information overload. Simple measures such as assessing concordance with inhaled steroids or simple track and trigger tools (analogous to ward-based ‘early warning scores’) are likely to be incorporated into practice in the near future once initial rules are resolved. The visualisation of such trends is currently technically feasible, but will also require standardisation. However, devolving greater responsibility to supervised artificial intelligence systems that can process large-scale parallel complex data (such as location) in near real-time, challenges the current nature of clinical practice and notions of physician responsibility. Without this change being properly managed, clinicians risk being overwhelmed with the amount of data they have to handle.[71]

## Conclusions

The transition from paper to electronic recording of medical data is progressing at pace, particularly where there are/were incentives for adoption and subsequent realisation of healthcare payment and reimbursement through adoption and usage. With the proliferation of EMRs comes complementary opportunities for care, resource efficiencies ​​​​ and clinical research. As we move forward, the major investment in EHRs and new technologies will not be in the hardware, but in change management as healthcare professionals learn how to use these digital tools and their data effectively, and providers restructure systems to cope with flexible, responsive, and personalised care.
